# Practicalities and challenges in re-orienting the health system in Zambia for treating chronic conditions

**DOI:** 10.1186/1472-6963-14-295

**Published:** 2014-07-08

**Authors:** Carolien J Aantjes, Tim KC Quinlan, Joske FG Bunders

**Affiliations:** 1 VU University Amsterdam, Faculty of Earth and Life Sciences: Athena Institute, De Boelelaan 1085, Amsterdam, HV 1081, The Netherlands; 2 ETC. Foundation, Kastanjelaan 5, Leusden, The Netherlands; 3 HEARDUniversity of KwaZulu-Natal, De Boelelaan 1085 Westville Campus, University Road, Durban, HV 1081, South-Africa

**Keywords:** Re-orientation, Chronic care, HIV, NCD, Health system transition, Model

## Abstract

**Background:**

The rapid evolution in disease burdens in low- and middle income countries is forcing policy makers to re-orient their health system towards a system which has the capability to simultaneously address infectious and non-communicable diseases. This paper draws on two different but overlapping studies which examined how actors in the Zambian health system are re-directing their policies, strategies and service structures to include the provision of health care for people with chronic conditions.

**Methods:**

Study methods in both studies included semi-structured interviews with government health officials at national level, and governmental and non-governmental health practitioners operating from community-, primary health care to hospital facility level. Focus group discussions were conducted with staff, stakeholders and caregivers of programmes providing care and support at community- and household levels. Study settings included urban and rural sites.

**Results:**

A series of adaptations transformed the HIV programme from an emergency response into the first large chronic care programme in the country. There are clear indications that the Zambian government is intending to expand this reach to patients with non-communicable diseases. Challenges to do this effectively include a lack of proper NCD prevalence data for planning, a concentration of technology and skills to detect and treat NCDs at secondary and tertiary levels in the health system and limited interest by donor agencies to support this transition.

**Conclusion:**

The reorientation of Zambia’s health system is in full swing and uses the foundation of a decentralised health system and presence of local models for HIV chronic care which actively involve community partners, patients and their families. There are early warning signs which could cause this transition to stall, one of which is the financial capability to resource this process.

## Background

Health systems in sub-Saharan Africa are under pressure to respond to demographic and epidemiological developments which are likely to increase the use of health care services, user health care costs, and operational costs for governments [1-3]. Health systems in this region have had to adapt continuously since the 1990s to a sequence of changing health care demands. During the 1990s, rampant HIV epidemics in many countries led to ever increasing demand for palliative care services which, in turn, led to the establishment of Home Based Care (HBC) programmes [4,5]. Anti-retroviral therapy (ART) programmes, established in the early to mid 2000s, expanded rapidly to reach an ever increasing number of people in need of treatment [6,7]. Key features of this process in the last 6–8 years have been decentralisation of HIV treatment and care to primary health facilities and diversification of HBC programmes to provide psycho-social support in various ways to those infected and affected by HIV and AIDS. HBC programmes evolved into Community and Home Based Care (CHBC) programmes oriented to providing comprehensive care and continuum of care services [8-15]. Now, the health care demand is to adapt the services that evolved towards provision of long term care for patients on ART, and to accommodate patients with other chronic illnesses such as hypertension, diabetes and cancer [16,17]. This article focuses on this nascent process. We discuss the process as health system transition; meaning re-orientation of health services to serve chronic conditions which, in due course, transforms the health system. We use developments in Zambia to illustrate this process.

Our broader aim is to move beyond the debate in the research literature about the potential for leveraging the ‘HIV service system’ to accommodate the projected increase of non-communicable diseases (NCDs) many of which require chronic care services [18-23]. In 2008, the World Health Organisation (WHO) advocated for combination of services for NCDs and communicable diseases such as HIV and TB [24] but, to date, there have been few publications which assess how this should work in practice [25-30]. We use research from Zambia to show how one country has moved beyond rhetoric and has begun to address the practical challenges of providing chronic care for patients with HIV and for patients with NCDs.

## Methods

The origin of this study was exploratory research in 2009 in which typologies of national HIV responses were constructed on the basis of semi-structured interviews with sixteen international health and HIV and AIDS experts using the technique of appreciative enquiry. These informants were selected, on the basis of a convenience sample, from the network of the first author. Results from that survey were then presented to twelve health and HIV and AIDS experts based in Uganda, Swaziland and South-Africa to verify whether the typologies resembled in-country responses to HIV and AIDS. That study helped to identify important components (such as decentralisation of health care services, multi-sectoral interventions and mobilisation of the patient population) of national HIV programmes which, at the time, had not been subject to extensive research and which informed the focus of two subsequent studies.

The first one was a comparative, cross-country study (Ethiopia, Malawi, South Africa and Zambia) conducted in 2011 and 2012 on the role of community caregivers in the expansion of HIV treatment services and in relation to primary health services [31-35]. Criteria for country selection included national government commitment to develop PHC services, the presence of a generalised HIV epidemic, and well established HBC/CHBC programmes. The research was conducted in several phases by in-country research teams. The researchers used a range of methods to identify past, present and emerging caregivers’ roles and enable triangulation of findings. Methods were used not only to provide an overview on the history of CHBC programmes but also to gain insight into adaptations over time by those involved in them. Methods included in-depth interviews, questionnaire surveys, focus group discussions, document review, and service observation and, for validation of findings, a final questionnaire survey in each country amongst CHBC service organisations, which was based on the common research results from the study as a whole. Generic interview schedules and field work procedures were used in each country.

Key informants included officials from Ministries of Health, Ministries of Community Development and/or Welfare, national AIDS coordinating bodies, large care and support organisations, PLHIV networks, and funders of CHBC programmes. The research includedin-depth case studies of three CHBC programmes in each country. The criteria for sampling were that the selection covered programmes which a) had been operational for more than 10 years, b) were managed by different organisations, c) were generally representative of CHBC programmes in each country, d) offered diversity of services in care (not exclusively health) as well as in their range of clients (HIV as well as other chronic illnesses) e) and that the sample included both urban and rural programmes. In order to minimise selection and information bias by the researchers, actual selection was facilitated by consultation with ‘advisory boards’ set up in each country for the study. These boards consisted of representatives of CHBC programmes, staff from national HIV programmes and community caregivers.

In each programme, semi-structured interviews and timeline exercises were conducted with staff members and external stakeholders (such as local clinic staff), as well as focus group discussions and community mapping exercises with caregivers, service observations and structured interviews with patients and their relatives. Data from interviews and focus group discussions were transcribed verbatim, and when FGDs were held in vernacular languages, scripts were translated into English. The analysis of this data was standardised using a structured coding model based on the study objectives and recurrent themes in the literature. The coding guide was distributed to the teams for input and compatibility checks and data subsequently entered into software programme Atlas.ti. The overall findings were used to compile a short questionnaire for distribution to 15 care organisations in each country (12 which had not been involved in the research and three to the managers of the programmes which had participated). Ultimately, 59 questionnaires were distributed and 46 were completed and returned. In addition, the country reports were presented and discussed with the advisory boards prior to public dissemination.

The second study is a continuation of the former study and was conducted by the first author from 2012 until early 2014. It examines the development of chronic care services in Zambia through information derived from government officials and care organisations on current and projected policies and plans for provision of chronic care.

Semi-structured interviews were conducted with 18 key informants operating at the national level, and 17 key informants operating at district- and primary health care level in the Zambian health system. In addition, 4 focus group discussions were conducted with key informants providing care and support at household level. The study was conducted in two of the three selected CHBC programmes which were sampled in the first study in Zambia and included one urban district (Lusaka) and one rural district (Mazabuka). All interviews were conducted in English, whereas focus group discussions were conducted in the vernacular languages and translated into English for analysis purposes. Data from interviews and focus group discussions were transcribed and coded using software programme Atlas.ti.

Key informants included officials from the Ministry of Health (MoH), Ministry of Community Development Mother and Child Health, National AIDS council, Zambian Network of people living with HIV, Church Health Association of Zambia, technical partners and funders of the Ministry of Health. Interviews were also conducted with district health authorities and with clinical staff of the attached health facilities, run in conjunction to two of the three selected CHBC programmes run by non-governmental organisations which were also sampled in the first study for Zambia. The research covered visits to 8 different level health facilities (from rural health centre to tertiary facility) as structured in the Zambian health system. A research assistant conducted interviews with an additional 19 key informants, which included among others MoH staff responsible for collecting and aggregating national health data, to obtain in-depth information on the functioning of the health management information system (H-MIS) and appropriate quantitative data. For the validation of the findings, summaries of all interviews were forwarded to the respondents for review and overall findings were verified via another series of interviews with a smaller sample of 8 national level key informants. Table 1 summarises the informant samples sizes from both studies.

**Table 1:** Informant samples of the two studies

**Research method and sample categories**	**Study 1 Zambia (4 countries; total sample size)**	**Study 2 Zambia**
Online survey among international experts	n/a (17)	n/a
Key national level informants in government and care organisations	13 (49)	18
Key informants CHBC programmes	8 (71)	17
FGD with CHBC programme staff	9 (17)	n/a
FGD with secondary caregivers in the CHBC programmes	29 (115)	36
FGD with community representatives in the CHBC programmes	20 (65)	n/a
Individual interviews with clients	30 (98)	n/a
Individual interviews with primary caregivers	30 (99)	n/a
Additional round of interviews with key informants on H-MIS	n/a	19
Validation Interviews: key informants at national level	5 (21)	8
Validation questionnaire survey: care and support organisations	9 (46)	n/a

The first study was granted ethical approval by the appropriate boards in all four countries. The second study received ethical approval from the biomedical research ethics committee of the University of Zambia (reference 013-02-11). Both studies were approved by the medical ethics review committee of the VU University Medical centre in Amsterdam, the Netherlands (references 2011/180; 2011/206).

The principal limitation for this article is the difficulty encountered in obtaining appropriate statistical data from the Zambian health management information system (H-MIS). Much of the purpose of building in an additional round of interviews in the second study was to find quantitative data which would help us to assess the strategic shifts in Zambia’s health system and the different perspectives of senior health officials on the needs and direction of these shifts. As indicated later in the article, there was limited data to assist our assessments.

## Results

This section presents findings from the two primary research studies. The results are presented in sub-sections as a means to delineate the adaptations of the Zambian health system since the early 2000s with reference, as necessary, to earlier developments. We highlight first the positive developments before drawing attention to gaps within, and limitations of the system.

### From emergency response to chronic care: Zambia’s HIV and AIDS programme

**Figure 1:**
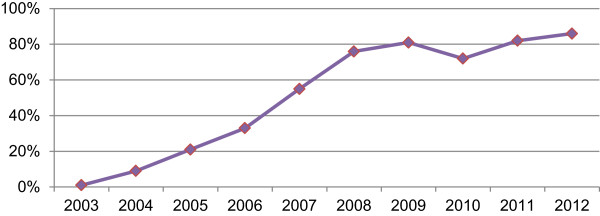
**Treatment coverage rates for Zambia.***Source: WHO/UNAIDS 2003-2012 estimates.*

Anti-retroviral therapy (ART) became available through the public health system in most Southern African countries, including Zambia, in the early to mid-2000s. In 2005, in Zambia, only 21% of patients in need of ART had access to it. By 2012, 86% of those in need were receiving treatment [36]. Table 2 and Figure 1 below summarise this remarkable progress towards achieving the goal of ‘universal access’ [36-38]. To illustrate, there are only five African countries with generalised HIV epidemics which were reaching this standard by 2012: Botswana (>95%), Namibia (91%), Swaziland (87%) and Rwanda (94%). Other countries in Southern Africa have lower coverage rates than Zambia: Malawi (76%), South Africa (81%), Lesotho (59%) and Mozambique (48%) and, generally in sub-Saharan Africa, 68% (6.9 million of 10.3 million individuals) of those in need of treatment were receiving it [36]. In Zambia, the principal challenges now are monitoring ART patients, retaining them in the treatment and support programmes, and treating treatment and HIV-induced morbidities. Enrolment of patients into these programmes (those assigned to receive ART and to pre-ART services) is the beginning of a long-term commitment for both patients and the health services.

**Table 2:** Treatment coverage rates for Zambia

**Year**	**Total number on ART**	Total number eligible for ART*	Total ART coverage**
2003	1100	220000	1%
2004	20000	230000	9%
2005	50000	230000	21%
2006	80000	250000	33%
2007	150000	270000	55%
2008	220000	290000	76%
2009	280000	350000	81%
2010	340000	480000	72%
2011	420000	510000	82%
2012	450000	520000	86%

*Data relating to the total number eligible for ART prior to 2010 were based on eligibility criteria for a CD4 count of less than 200. Meanwhile, the data presented from 2010 onwards were based on eligibility criteria for a CD4 count of less than 350. **The percentages refer to the share of all eligible for ART who were receiving it.

Source: WHO/UNAIDS rounded off estimates 2003–2012.

The Zambian HIV programme effectively changed from being an emergency response into a chronic care programme for several reasons. Firstly, the Zambian health system had a solid foundation for accommodating the changing needs of people living with HIV (PLHIV) as a result of:

a) The health system’s decentralisation strategy of the 1980s, which coincided with the beginning of the HIV pandemic in the country. Primary health facilities were established (e.g. health posts) as well as mechanisms to engage communities (e.g. neighbourhood health committees) [31,39,40]. This strategy was inspired by the Alma Ata principles of 1978 [41].

b) The rapid development of HBC services in late 1980s and the 1990s, which included NGO/FBO run health services in parallel to government health services (e.g. mission hospitals) [4,12,31].

c) The elaboration of HBC into community home based care (CHBC) as the ART programme expanded, and as NGO and FBO care organisations diversified their services to serve the changing needs of PLHIV [31].

d) The extensive co-ordination of activities of staff at PHC health facilities, community agents, such as neighbourhood health committees and community volunteer caregivers, and the managers and supervisors of the NGO and FBO-driven community health and social welfare programmes. This co-ordination enables patients and families to access economic, spiritual and social support, access to medical care and ongoing care and support as necessary [31].

Secondly, this foundation enables the implementation of the PHC revitalisation agenda advocated by the WHO and to which the Zambian government signed its commitment in 2008 [42]. Since then, the government has expanded PHC services through reinstating health posts, recruiting additional nurses, creating state-paid community health workers called Community Health Assistants, and beginning to draw NGO and FBO CHBC programmes into the orbit of responsibilities of PHC facilities (emphasising community ‘ outreach’ and disease and treatment surveillance). As a result, PHC facilities generally, in collaboration with community structures, provide a comprehensive package of health promotion, prevention (voluntary counselling and testing, prevention of mother to child transmission and screening of sexually transmitted infections), treatment, and monitoring (CD4 count, liver and renal functions, and full blood count). Again generally speaking, clients can access (adherence) counselling services, medical consultation with either a nurse of clinical officer, and first line ART at this level. PHC facilities have the capacity to treat opportunistic infections, while complicated infections are referred to a hospital. However, as we discuss later, we discerned marked variation in the service capacities of PHC facilities but were not able to quantify via H-MIS data. The decentralisation of HIV related services to PHC level has been accommodated by task shifting. Examples include treatment prescription extended from doctors to specially trained nurses and counselling services, formerly executed by nurses, completely shifted to lay counsellors. The Ministry of Health (MoH) now also offers a nurse practitioner course in the country, who perform additional tasks in treatment management besides prescription, and informants in our study suggested allowing midwives and TB officers to prescribe ART as a future option.

Thirdly, the country has been a recipient of large amounts of dedicated funding for health system strengthening and HIV interventions since the mid-2000s [6,43]. Funding increased dramatically in 2004 when PEPFAR started disbursing funds to Zambia and by 2006, the amount dedicated to HIV and AIDS programmes almost equalled total health budget per capita [44]. According to the informants, this funding has enabled training of health professionals, building the physical infrastructure and support systems (e.g. introduction of new laboratory technology at lower level health facilities such as CD4 count, liver and renal function testing), and medicine procurement.

Fourthly, the Ministry of Health has received substantive external technical assistance according to the research informants. In the mid 2000s, this included training in ART management for MoH health staff (a training which is now offered by the MoH itself), training for laboratory staff, assistance in setting up supply chains for ART drugs, and the implementation of an electronic monitoring system for patients on ART (mainstream health services still work with paper-based files) since 2006. More recently, technical assistance included support to the development of an advanced treatment centre with highly specialized care for PLHIV on ART experiencing severe complications, drug adverse reactions or drug resistance and experimentation with the integration of ART services into the out-patient departments of selected health facilities in Lusaka- and Southern Province [45].

**Figure 2:**
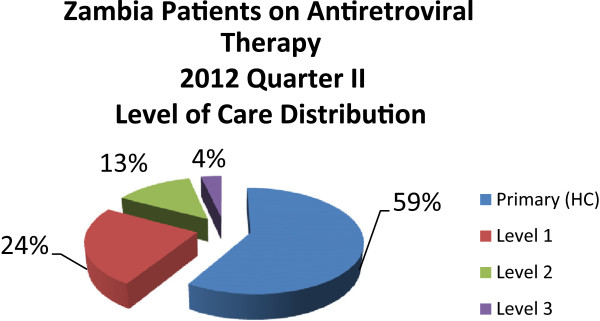
**Distribution of ART patients across health system levels in Zambia.** Source: Database MoH, 2012.

Table 3 summarises the infrastructure and extent of services that the Zambian public health system can provide now for PLHIV. It should be noted that we refer here to both government and NGO/FBO infrastructure.An indicator of the value of this infrastructure is the extent to which the ART programme has been decentralised. The majority of those on treatment, including children, access the ART at PHC level as is indicated in Figure 2.

**Table 3:** Service structures at the different health system levels for PLHIV in Zambia

**Location and actors**	**Services**
Tertiary level hospital (specialists)	One advanced treatment centre in Lusaka with specialised doctors who can treat patients with third line ART, address serious complications, screen for treatment resistance and one specialised Psychiatric hospital for serious complications in mental health
Second level hospital (doctors and some specialists)	Range of diagnostic, medical care and treatment for PLHIV, management of complicated cases from the first level hospitals and PHC level
First level hospital (doctors and clinical officers)	Provision of ART first and second line treatment, attending to complications from PHC level, PCR testing specimen investigations, not sufficiently equipped to address mental health problems of patients
Primary health care facilities (nurses and clinical officers)	Health promotion and prevention activities on HIV /AIDS, voluntary counselling and testing, prevention of mother to child transmission, screening of sexually transmitted infections, provision of first line ART, treatment of opportunistic infections, adherence counselling and in some districts provision of mosquito nets and water filters for PLHIV
Community level (community caregivers, community health assistants, neighbourhood health committees)	Community Home based care for bedridden patients, follow-up visits to promote treatment adherence, nutritional advice, counselling, both spiritual and psycho-social, referrals, economic support, instructions to family members on caring for their HIV infected relative. Task of CHAs is mainly on prevention, basic hygiene, referral to health facility if needed
Household level (patients, family members)	Relatives function as treatment buddies, peer patients may also take up this function, availability of support groups for PHIV

**Figure 3:**
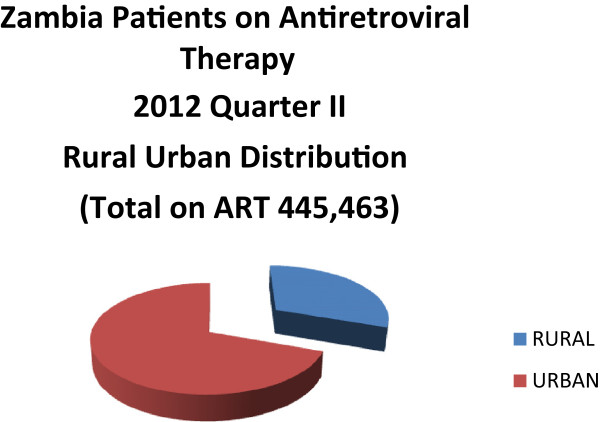
**Distribution of ART patients across urban and rural health facilities.** Source: Database MoH 2012.

Nonetheless, there are also indications that the system is struggling to accommodate relevant demographic factors. Figure 3 summarises MoH data, showing that 75% of all ART patients access treatment from urban health facilities. There has been a 4.2% population growth rate in urban areas compared to 2.1% in rural areas between 2000 to 2010 [46]. However, a majority (65%) of the population still resides in rural settlements [47] which suggests, as Figure 3 infers, that Zambia’s high general ART coverage rate obscures relatively low coverage rates in rural areas. In addition, in 2010, only 25.4% (453) of an official estimate of 1,784 health facilities in the country reportedly provided ART [48]. Add also an MoH assessment that 99% of urban Zambians live within five kilometres of a health facility, compared to only 50% of rural residents [49] and the inference is that the latter have less access to ART than the former. We emphasise that this is a provisional analysis circumscribed by lack of data; notably, reliable statistics on the proportions of urban and rural people living with HIV, and the spatial distribution of health facilities linked to population catchment area and population size in each area.

### Emerging challenges: dealing with HIV and non-communicable diseases

It is predicted that in 2030 non-communicable diseases will be the most common causes of death in the African region [50,51]. Estimates of the NCD mortality rates in Zambia are high with 1075 male deaths and 808 female deaths per 100,000 habitants in 2008 [51]. In comparison, the overall NCD mortality rates in low- and middle-income countries in 2008 were 756 per 100,000 for males and 565 per 100,000 for females. These are respectively 65% and 85% higher than in high-income countries [51]. In recent years, studies have started to report on the association between HIV infection, HIV treatment and NCDs [25,52-56]. Up to date, there has not been a study in Zambia which reports on this association. Clinician informants of our second study, working directly with ART patients, reported that they are encountering NCDs in these patients. The most common co-morbidities are hypertension, diabetes, stroke and cervical cancer. These informants also suggested that the rise in NCDs among ART patients may be as a result of a growing economy and changing lifestyles. In contrast, informants providing community care to patients on ART, such as volunteer caregivers and peer patients, did not mention NCDs frequently as a challenge. In response to questions on client health complications, they highlighted the side-effects of treatment for clients who had been stable on ART for at least 5–7 years. Community caregivers generally conveyed a message of their clients being in good health, now that they are on ART. The majority of Zambians enrolled in ART programmes are on a first line regimen but patients, who experience severe complications as a result of co-morbidities or patterns of treatment resistance, can be referred to the advanced treatment centre in the capital. This centre has trained clinicians for treating HIV and treatment-related NCDs and can authorise change in ART up to ‘third line’ regimens. Reportedly, the MoH intends to establish similar centres in each province of Zambia.

The anecdotal evidence reflects increasing official attention on NCDs. A number of national policies formulated within the last three years, address the emerging burden of NCDs. They include the national health policy, which was passed by Cabinet in 2012, and the 2011–2015 national health strategic plan which consider NCDs as one of the health priorities [49,57]. Reportedly, a new ’Health Act’, still in development, will address NCDs explicitly. These policies and legislation indicate the route that the Zambian government is taking. The national health policy, for example, is aligned with national community and economic development policies [58] and seeks to provide all Zambians with quality health care “as close to the family as possible” [57]. This implies commitment to provide integrated disease treatment and management at the level of primary health care. One indicator of this commitment is the recent transfer of all PHC operations from the Ministry of Health to the Ministry of Community Development and Mother and Child Health (MCDMCH) in 2013. Nonetheless, the MCDMCH will use a NCD strategy and updated clinical guidelines for different conditions that were devised by the MoH in 2012. The strategy and guidelines were reportedly compiled via a collaborative process involving representatives of relevant government and civil society agencies, using the HIV service system as a foundation. The strategy outlines the operational adaptations which need to be made to improve service delivery at all levels of Zambia’s health system and awaits approval from the Minister of MCDMCH and the WHO. It is important to note that our study exposed a lack of clarity about the actual approach to provide integrated disease management at PHC level. Two agendas seemed to exist at senior policy level, with one being the agenda to incorporate NCD care into the existing ART clinics and another one to incorporate ART services into general out-patient departments.

Currently, the evident challenge is the limited availability of appropriate health care services for people with NCDs at the lower levels in the system. NCD diagnosis and treatment are incorporated in the training curriculum of every medical doctor, clinical officer and nurse in Zambia but clinician informants reported limited delivery of NCD services. The bulk of NCD diagnosis, treatment and monitoring is currently conducted at secondary and tertiary level facilities.

The main tertiary facility in the country is the University Teaching Hospital (UTH) in capital Lusaka. Patients are attended to by consultants (specialist clinicians) in various departments which have technology such as MRI, CAT and CT scans at their disposal. The hospital also has a cancer hospital on the premises. The cancer hospital is the only public hospital where chemo- and radiotherapy is provided in the country. Other tertiary care facilities in the country are the Atta Davidson Children’s hospital in Ndola and the Psychiatric Hospital Chainama in Lusaka. A senior clinician at UTH articulated the current challenges in the management of NCDs as follows: ‘*the very fact that a diabetic would develop kidney complications, the very fact that they will develop retinal complications or eye complications, the fact that they will end up with an amputation means that the management is not up to scratch’.* According to him, hospital staff is always ‘fire-fighting’ cases which could have been prevented if there was effective monitoring of NCDs in patients at primary and first (district) level facilities. Another clinician at the national MoH office stated that it will take time to establish this mechanism: ‘*we are looking at getting the clinical officers and the nurses into the game because NCDs have been mainly managed by doctors and that has been a limitation for its decentralization […] I do not feel we will be able to do it in the next two years; to actually do everything at the health centre or ward’*.

Some general (secondary level) hospitals have necessary equipment such as mammography and other imaging devices. CT scans are currently only available in the secondary and tertiary hospitals in Lusaka and in some private sector facilities but, reportedly, the MoH is procuring more of these items for placement in provincial hospitals. All secondary hospitals can treat hypertension, cervical cancer and diabetes patients who require insulin. However, these facilities have limited means to address obesity and cholesterol-related illnesses. Cholesterol medication is not on the essential drug list and there is a shortage of specialist professionals such as nutritionists. Again, however, these limitations have been acknowledged and the MoH intends to establish specialist NCD clinics in every provincial hospital to provide services for cardio-vascular illnesses, hypertension, diabetes and cancers. The clinics are to be staffed with specialist clinicians, either as a permanent staff or as ‘consultants’ who conduct periodic visits to the hospitals. At district (first level) hospitals, diabetes, hypertension and some forms of cancer (depending on the available equipment) can be diagnosed. Treatment of conditions such as hypertension and diabetes can be initiated at this level. Clinical officers can also manage clients with uncomplicated cases of diabetes and mild to moderate hypertension, perform dip treatment for cervical cancer, and minor surgery for diabetes complications. Patients with more serious complications may be put on a waiting list for the periodic visit by a medical doctor. Health staff can also monitor blood pressure, sugar levels and do review for asthma, either during a clinical consultation or through admission for observation in the hospital.

At the PHC level, there is a limited capacity for NCD treatment. Generally, informants reported common challenges with drug procurement, limited availability of diagnostic equipment, and of different medicines to treat the range of NCDs. For example, reportedly, urban health centres usually have oral medicines for diabetes but not all rural health centres. Current screening and monitoring services for NCDs include blood pressure measurement, other general vitals such as height and weight, and urine dipsticks. The finger prick method for informing an accurate treatment dosage for diabetic patients is generally not available. According to informants working at this level, monitoring of the conditions occurs more often at the initiative of the patient than the health provider with follow-up appointments not being a standard procedure.

In 2012, the MoH introduced Community Health Assistants (CHAs). CHAs are to serve as community health workers and are the lowest level of officially registered health professionals. They receive a year’s training and are deployed to health posts to provide promotive and preventive health activities (such as disease surveillance) in the community. The current training curricula of the CHAs include a disease focus on HIV, malaria, respiratory illnesses, diarrheal diseases and TB. However, there are plans to include health promotion and the screening of hypertension and diabetes into the CHA training curriculum and their job descriptions. It may be noted that CHBC programmes had previously begun to assist people suffering from NCDs in the course of diversifying their care and support services, though via volunteer caregivers who had no formal relevant training in this area [31].

In summary, at present, there is a clear disparity in scale and reach of HIV and NCD services. HIV services, including ART and management of opportunistic infections, have been decentralised to PHC level thereby showing the ability of the system to deliver ‘quality health care as close to the family as possible’. NCD services are restricted and have yet to be decentralised. Patients have to go to hospitals and, probably in many cases, to distant secondary level facilities and even to the tertiary facilities in Lusaka to obtain treatment in view of limited distribution of the necessary clinician skills and technologies in the country’s hospitals.

### Monitoring the burden of disease of chronic conditions in Zambia

The gaps in the current state of the NCD services are also evident in the limited scope for monitoring the incidence and prevalence of chronic diseases. Data on HIV and AIDS are consistently collected through the country’s H-MIS which is supported by data retrieved from a country-wide electronic system tracking HIV positive patients enrolled in treatment services. This system, called Smart-care, captures and stores individual patient information on a chip card that is provided to the patients. Information includes the treatment regimen, number of visits to the health centre and laboratory test results. In 2013, the MoH piloted the use of this system in one PHC clinic to all patients visiting this facility. Non-routine data on HIV is also captured by on occasion Zambia Demographic and Health Surveys and Zambia HIV/AIDS Service Assessments. The national picture on the occurrence of chronic diseases in Zambia cannot easily be extracted from the H-MIS for several reasons. Firstly, only patient data from PHC facilities, district and general hospitals is linked to the H-MIS. This data is supplied through handwritten reports to the district, where the data is entered into an electronic system. The patient data from tertiary hospitals and private health care facilities is not routinely linked to the H-MIS. As of 2013, the Ministry of Health commenced the roll out of a hospital based H-MIS at tertiary level to address this gap. Secondly, there is limited specification of types of chronic diseases recorded at facility-level: disease specification is recorded for HIV, diabetes, hypertension, asthma, epilepsy sickle cell disease, cervical and breast cancers but general categorisation for other cancers, mental disorders and cardio-vascular diseases. Thirdly, the Zambian H-MIS is predominantly a paper-based system, which hampers the timely extraction of data at national level.

**Figure 4:**
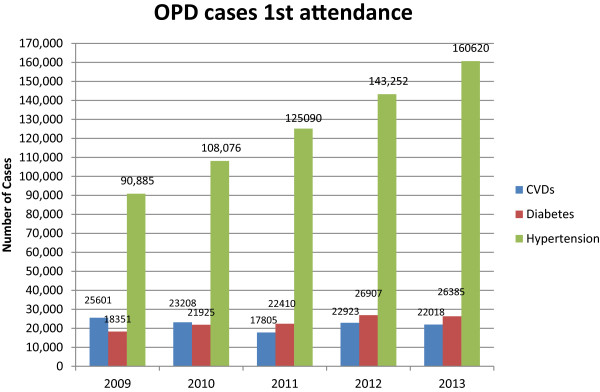
**NCDs presenting at OPDs in Zambian health facilities.** Source: H-MIS,MoH, 2014.

**Figure 5:**
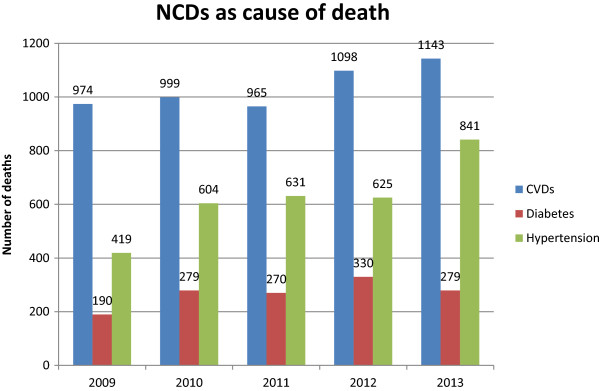
**Mortality rate of NCDs as reported by Zambian health facilities.** Source: H-MIS,MoH, 2014.

During the second study, we retrieved data from different sources in an attempt to provide a preliminary assessment of the burden of non-communicable diseases in Zambia. One source was the ‘STEPS survey’, which used the WHO’s STEP-wise approach to surveillance of NCDs. This first large-scale NCD survey in the country commenced in 2007 and was still being carried out in 2014 by the MoH. Findings from one district (Lusaka district) have since been published [59-62]. The findings from a larger sample of four districts, still unpublished, suggest that 28.0% of the country’s women and 17% of the men suffer from hypertension (MoH, 2012 personal communication). These preliminary findings are lower than the WHO estimates of 2008: 37.9% age-standardized prevalence rate of hypertension in women and 41.0% in men [37].Another source was the H-MIS. With the support of MoH officials, we extracted and analysed national data on incidence of hypertension, diabetes and cardio-vascular diseases from the health facilities which are linked to H-MIS. The results are presented in Figures 4 and 5 below. The data represented in Figure 4 shows that there has been a steady and marked increase in the number of hypertension cases reported at PHC facilities but no increase in the number of reported cases of cardio-vascular disease (CVDs) and diabetes. Figure 5 shows that, except for hypertension, there have been marginal increases in NCD-related mortalities.

We emphasise that limited use can be made of the data. Firstly, the data cannot be used to estimate NCD prevalences as the denominator is not provided in the H-MIS. Secondly, this data is not complemented by statistics from tertiary level hospitals which, according to our research, receive a substantial amount of NCD cases, nor by statistics from private health facilities. Thirdly, the system does not track repeat visits and, therefore, there may be double counts in the data sets. Fourthly, there is reason to doubt the accuracy of the data collection at health facilities in view of the heavy workloads of staff and the extensive details and time required to complete the H-MIS forms.

In summary, there is currently no reliable information of the incidence and prevalence of NCDs in Zambia. Nonetheless, chronic care services are work-in-progress though with limited scope for informed planning; for instance, as one senior MoH informant explained, the calculations for procurement of NCD medicines are based currently on consumption data.

## Discussion

The Zambian case illustrates the opportunities and the challenges of re-orienting an under-resourced health system to include chronic care services. A re-orientation of the Zambian health system was achieved for PLHIV: from a stand-alone emergency programme to a chronic care programme with strong linkages between the different levels within the public health services and between them and other government ministries and civil society care organisations. A further re-orientation is occurring now as the HIV and AIDS care programme is used as the basis to address NCDs.

Our research shows that the current process is one of necessity and pragmatism. Interviews with a range of senior health officials did not indicate use of any specific theoretical model on chronic care, devised by scholars or international agencies, but it is possible to discern elements of different models such as the Chronic Care Model (CCM) by Wagner and the WHO’s Innovative Care for Chronic Conditions (ICCC) Framework [63,64]. The CCM delineates commonly accepted key elements of chronic care services. It highlights the structural components (‘Community’ and ‘Health System’) which need to co-exist for effective delivery of the services (the ‘productive interactions’) which, in turn, allow for beneficial effects (‘improved outcomes’). Table 4 summarises the current state of Zambia’s chronic care services for HIV and NCDs in terms of those key elements. It should be noted that ‘self-management’ may be defined as “learning and practicing skills necessary to carry on an active and emotionally satisfying life in the face of a chronic condition” [65]. In this, the involvement of family members, community partners and health care providers is imperative [63].

**Table 4:** Comparison between adaptations made to the health system for patients with HIV and NCDs

**Health systems**	**HIV**	**NCD**
Delivery system design	Clinical HIV services have been decentralised to enable care and treatment at PHC level. There is division of tasks between different disciplines. Higher levels facilities attend to complicated cases and include recent introduction of an advanced treatment centre. Intention to establish such centres in each Province.	Clinical care for NCDs is suboptimal at PHC level. Higher level facilities predominantly manage uncomplicated and complicated cases Very limited specialist infrastructure (e.g. one Cancer hospital) Intention to establish specialist centres at all provincial hospitals.
Decision support	Established ART guidelines and trained personnel (including NGO/FBO caregivers) which provides capacity for patient surveillance, recruitment, adherence and retention: enables early detection of complications (HIV and ART-induced NCDs).	Updated treatment guidelines not yet released. Limited NCD care expertise at PHC level such that patients are referred to higher levels (where there is shortage of specialist health professionals).
Clinical information systems	Electronic information system being installed at PHC facilities (will improve patient monitoring, quality of service and patient medical data). Relatively reliable data for assessment of HIV incidence and prevalence but not yet for NCD co-morbidities.	Paper-based information system and lack of NCD specification in H-MIS data collection. NCD specification indicators to be added to tertiary hospital H-MIS data collection. Technical constraints prevent inclusion of NCD data on HIV electronic information system Smart care.
**Community**	**HIV**	**NCD**
Self-management support	Decentralisation of ART and introduction of electronic information system creates scope for greater individual –focused care. Self-management concept well established via the diverse care and support services of CHBC programmes.	Some CHBC programmes have extended support to NCD patients but lack financial support and training. Self-management principles for NCD patients not yet included in CHA skills training.
**Outcomes**	**HIV**	**NCD**
Productive Interactions	CHBC programmes provide foundation for HIV literacy in communities, care and treatment skills amongst families and via volunteer caregivers. Revitalisation of PHC is expanding capacity and scope of PHC facilities for co-ordinated interventions with CHBC programmes.	Limited NCD literacy amongst the population exacerbated by lack of care and treatment skills amongst CHBC programmes and PHC health professionals. Suboptimal infrastructure for linking service provision between different levels of care (primary up to tertiary level) and between different disciplines.
Improved outcomes	Zambia achieved ‘universal coverage’ of ART (>80% coverage).	Lack of data on efficacy of NCD services (e.g. disease monitoring, appropriate referral).

The WHO framework is an expansion of the CCM and emphasises the importance of a positive policy environment and investment in service mechanisms at PHC level facilities. It was formulated and broadcast in 2002 for global use. However, there is no evidence that it has been used in Zambia; few health officials had heard of the model and none had any knowledge of its application in health policy discussions or in MoH strategies. It seems to have been little used elsewhere in Africa [66-68]. Our research results lead us to concur with researchers who have argued that the available and commonly cited models - all of which stem from experimentation and application in the ‘north’ [69]- are of limited use in Africa because they presume the presence of medical technologies, adequate numbers of skilled professional staff, and well-developed health systems [25,28].

This is not to say that chronic care services are not evolving in sub-Saharan Africa. Our results direct attention beyond current debates about leveraging HIV programmes to establish chronic care programmes [18-23]. That leveraging is happening in Zambia on the basis of well-established CHBC programmes and the government’s interventions to revitalise PHC which now include restructuring of different ministries responsibilities to enable co-ordination of the activities of different agencies involved in this field of health care. Similar mechanisms exist elsewhere in Africa [34] such that it is perhaps appropriate to observe the emergence of an ‘African’ chronic care model.

However, we have drawn attention to the fact that the Zambian health system is in transition and, hence, working through a host of practical challenges with regard to building NCD services on the basis of existing HIV service structures. A lack of clarity at policy level on how to approach the integration of services at PHC level has not prevented innovative experiments [45,70], but substantial further efforts are required to capitalize on the (primary care) foundations of the HIV programme. Existing linkages between CHBC programmes, CHAs and PHC facilities can support the early detection, screening and appropriate referral of NCD patients within a broader remit of chronic care and treatment services in the country. This requires investment, funding and (re-)training of health professionals and community caregivers in addition to the investment in specialist services at the first, secondary and tertiary level facilities. Currently, there is little indication that international donors will support this investment, particularly in community-level structures [71].

**Figure 6:**
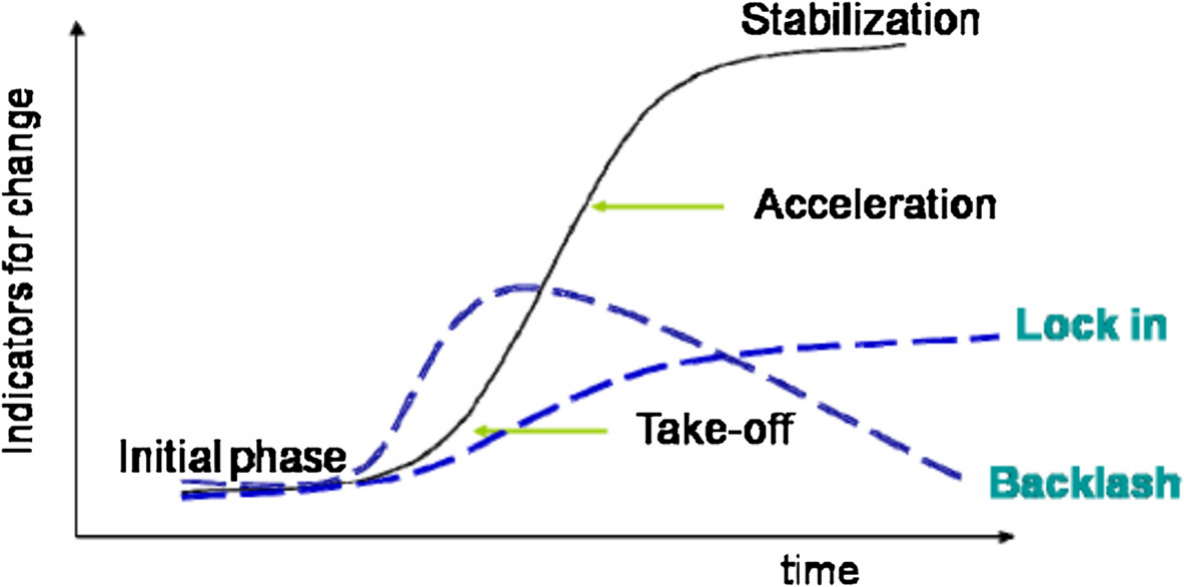
**Transition pathway.** Source: Loorbach, 2007.

The Zambian health system is in transition but there is no certainty of the outcome. Figure 6 outlines theoretically the different possible outcomes for health systems in transition [72,73]. This model is based on the premise that health systems are complex, adaptive systems consisting of a multitude of actors and subsystems within the system. National health systems evolve continuously in response to the changing burden of disease and health service needs of the population, and efforts (of the many actors) to improve the quality and equity of the health services. However, the actual outcomes depend on whether actors make the necessary modifications to dominant structures, cultures and practices within a health system that will enable change [74-76]. Briefly stated, changing health conditions in a country stimulate innovation which is represented in terms of ‘pre-development phase’ (the point where need for change is acknowledged) and the subsequent trajectories of interventions, all of which begin with the intention to adapt the health system constructively. However successful adaptation, meaning system changes that are sound and effective in meeting the changing burden of disease and health care demands (and represented by the trajectory for ‘take-off’ to ‘stabilisation’ of the modified system at some point) is not the only possible trajectory. Innovation may lead to contests between actors within a system (e.g. politics of redistribution of authority and resources) or prove not to be sustainable for various possible reasons (e.g. financial, system re-design faults) which lead to efforts to prevent system change (represented as ‘backlash’). Alternatively (represented by ‘lock-in’) , initial innovation succeeds in generating some changes that are effective but the actors perceive a limit to what is required and do not follow through the innovations [74,77].

In the case of Zambia, innovation has occurred with the modifications of the HIV programme to accommodate ART and the changing health service needs of PLHIV and, currently, use of that programme as a basis to develop chronic care services. At present, there are indicators of further innovation (‘to provide quality care as close to the family as possible’) in view of the strong foundation for realising key components of chronic care services (notably community and family involvement in patient ‘self-management’) and overt commitment of the government (including shifting responsibilities for PHC operations from the MoH to MCDMCH). Equally, there are factors which could confound the initiatives, for example, possible contests of authority between the MoH and MCDMCH or between the government and civil society on regulation of NGO/FBO care organisations, inadequate funding for CHBC programmes to sustain a wide range of care and support services or delayed realisation of capacity (staff and technologies) for NCD screening, monitoring and treatment at lower levels in the system due to limited funding.

## Conclusion

Health system adaptations successfully resulted in a large scale chronic care programme for HIV in Zambia. Further adaptations to provide similar scope of services to patients with NCDs are underway, such as establishment of more facilities for specialist NCD care and intentions to include NCDs in the training curriculum of CHAs. This article has shown that there is strong potential to make the transition to a health system capable of managing the current and future health care demands for NCD and HIV chronic care by building on to the foundations of the current programme for HIV.

The Zambian case furthermore demonstrates that the issue in Africa now is not whether HIV programmes can serve as the foundation for introducing NCD programmes but whether recent and current system adaptations to address the needs of both HIV and patients with NCDs are sound. Our research indicates presence of sound principles and practices, for example the inclusion of ministries (which can address the social determinants of health) alongside the MoH in the management and organisation of services, the clarity of the role of the MoH to provide medical care and CHBC programmes to provide support to both patients and PHC facilities and the gradual establishment of facilities to handle chronic care. However, there are warning signs of where and why this transition could stall, for example, the little scope for informed strategic and operational decision making in view of the lack of evidence on the disease burden of NCDs in Zambia and the concentration of technology and skills at the second and tertiary levels in the health system. There is good reason to doubt whether there will be sufficient international donor and national government support, which was a feature in the HIV programme, to decentralise NCD service technology and skills to the primary level and to develop the electronic technology for monitoring a projected large and increasing number of people with NCDs.

## Competing interests

The authors declare that they have no competing interests.

## Authors’ contribution

CA and TQ conceived the studies and developed their methodology. CA and in-country research teams conducted fieldwork and interviews, with technical support from TQ and JB. CA, TQ and JB contributed to the interpretation of the findings. CA drafted and wrote the manuscript. All authors commented on different versions of the manuscript, and read and approved the final manuscript.

## Pre-publication history

The pre-publication history for this paper can be accessed here:

http://www.biomedcentral.com/1472-6963/14/295/prepub
